# Systemic versus Pulmonary Bleeding in Lung Infection: Value of CT in Two Cases

**DOI:** 10.5334/jbsr.1838

**Published:** 2019-08-16

**Authors:** Alexandre Kadou, Nicolas Verbeeck, Kamal Abou Hamdan, Catherine Charpentier, Alain Nchimi

**Affiliations:** 1 CHL, LU

**Keywords:** hemoptysis, bronchial artery aneurysm, pulmonary, computed tomography, angiography, embolisation

## Abstract

Bronchial and pulmonary artery aneurysms are rare causes of hemoptysis in the course of lung infection, for which early diagnosis and treatment are essential to prevent fatal bleeding.

Depending on patient condition, these occurrences are amenable to computed tomography (CT) to determine both the cause of hemoptysis and the bleeding site in order to plan the most effective bleeding-control procedure.

In this report, we illustrate the importance of the bleeding site identification using CT in two cases of infection-related hemoptysis.

## Introduction

Hemoptysis is the expectoration of blood or blood-tinged sputum from pulmonary parenchyma or airways. Life-threatening hemoptysis may cause abnormal gas exchange and haemodynamic instability, with a 50–80% mortality rate if left untreated [1,2].

There are numerous etiologies for massive hemoptysis, among which the most frequent are bronchiectasis, infection, and cancer. The rupture of arteries within airways may occur on both bronchial and pulmonary arteries, which determines a different vascular access for a bleeding-control procedure. In 90% of cases, the source of bleeding is from the bronchial circulation; in less than 10% of cases it is from the pulmonary circulation. Computed tomography (CT) has established itself as a standard diagnostic procedure to determine the cause and the localization of hemoptysis. CT therefore potentially provides information of paramount importance regarding patient management.

We present two cases of hemoptysis during the course of lung infection including a ruptured bronchial artery aneurysm (BAA) and a ruptured pulmonary artery aneurysm, the so-called Rasmussen aneurysm (RA), both successfully identified on CT before treatment by percutaneous embolisation.

## Case 1

**Figure 1:**
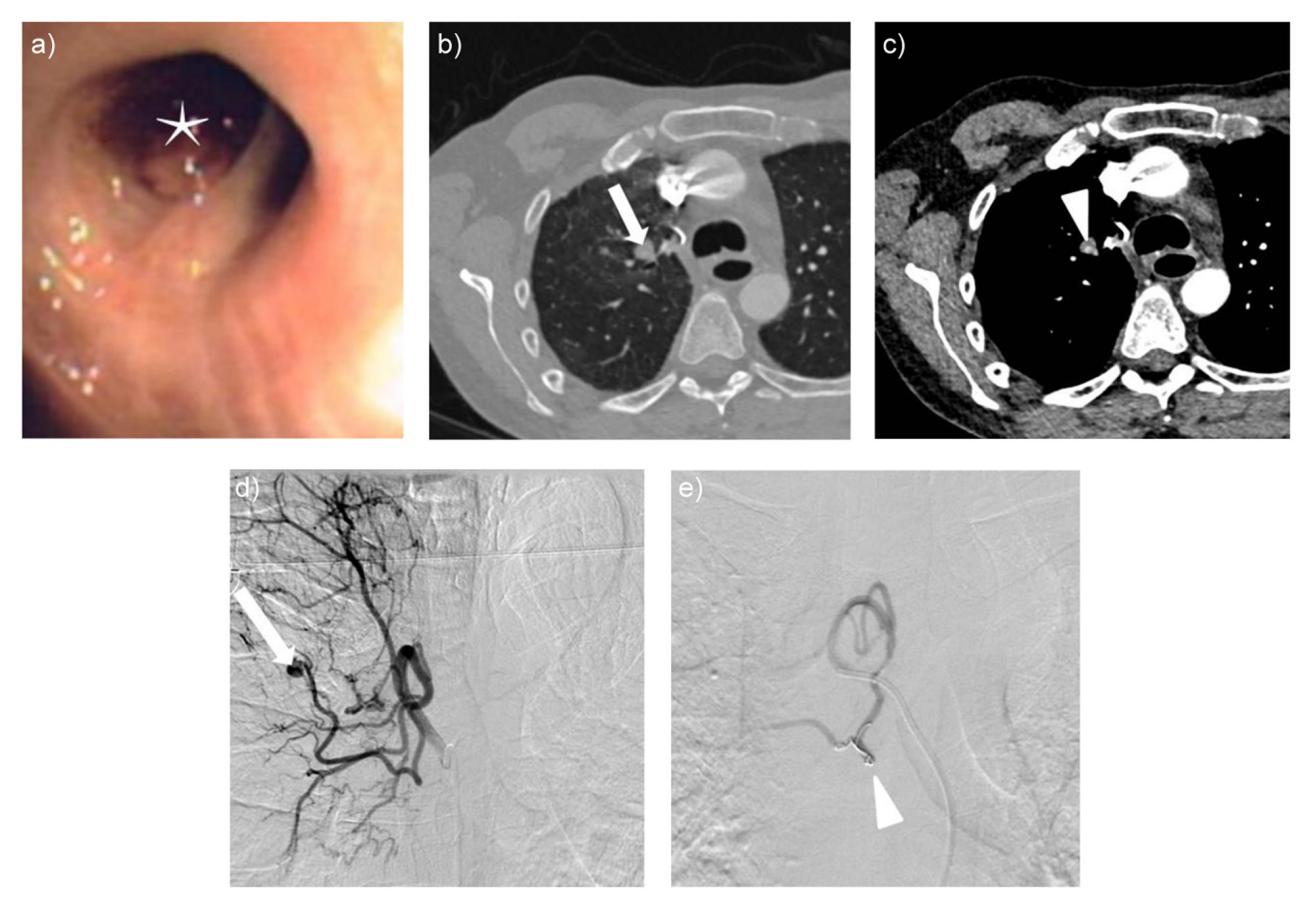
**a)** Bronchoscopy showed an organized blood clot obstructing a subsegmental bronchus of the right apical segment. **b)** and **c)** CT angiography showed bronchial obstruction at the level of the bifurcation of the apical and dorsal bronchial segments of the right upper lobe (b, arrow), along the corresponding arteries and containing a tiny (4 mm) contrast enhancement focus (c, arrow head), suggesting a BAA. **d)** and **e)** Digital subtraction angiography after artery catheterism showed the BAA in the right upper lobe (d, arrow). Selective embolisation was performed with microcoils (e, arrow head).

A 44-year-old man with a two-week history of massive hemoptysis, fever, and cough presented to our institution. A diagnosis of pneumonia was made, with right upper lobe lung consolidation on CT. The symptoms subsided after antibiotic therapy (amoxicillin and clavulanic acid). Following a relapse of hemoptysis, the patient was urgently re-admitted. Bronchoscopy showed a recent blood clot obstructing a sub-segmental bronchus of the apical segment of the right upper lobe (Figure 1a). Contrast-enhanced CT (80 mL of iodine contrast at the rate of 3 mL/s; the CT start after enhanced >100 HU in the ascending aorta; 0.6 mm collimation) showed a 4 mm spherical contrast-enhancement within the clotted bronchus suggesting a BAA (Figure 1b, 1c). Bronchial angiography confirmed the CT findings, and selective embolisation of the feeding artery with microcoils was successfully performed (Figure 1d, 1e). Patient recovery was uneventful.

## Case 2

**Figure 2:**
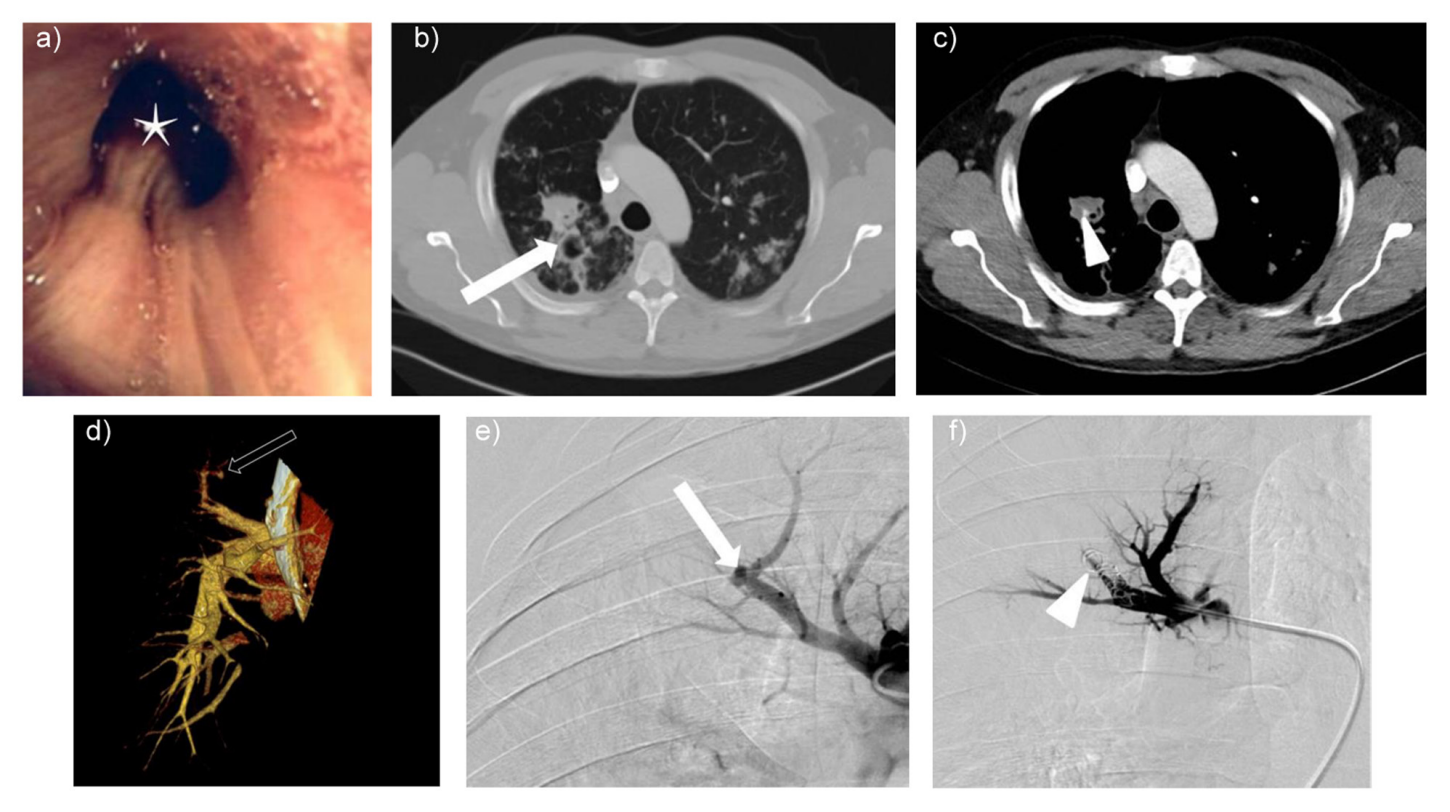
**a)** Bronchoscopy showed a clot in the right upper lobe bronchus. **b)**, **c)** and **d)** CT angiography showed consolidations and a peribronchial nodule at the level of the apical segment of the right upper lobe (b, white arrow) and a 7 mm contrast-enhancing focus (c, arrow head) originating from the right pulmonary artery, as visualised on volume rendering reconstruction (d, black arrow), suggesting an aneurysm of the pulmonary artery. **e)** and **f)** Digital subtraction angiography after pulmonary artery catheterism showed the aneurysm in the right upper lobe (e, arrow). Selective embolization was performed with microcoils (f, arrowhead).

A 38-year-old male was admitted for hemoptysis of average abundance (a little less than 100 mL in 24 hours) that occurred suddenly at his workplace. He was clinically in good health, showing normal vital signs. He was current smoker (23 packs/year) and had a history of exposure to a work colleague with tuberculosis. Bronchoscopy revealed a clot in the right upper lobe bronchus (Figure 2a) and bronchoalveolar lavage showed acid-fast bacilli. The Quantiferon dosage was 2.91 IU/mL, and the tuberculin antigen was positive. Contrast-enhanced CT (100 mL of iodine contrast at the rate of 3 mL/s; the CT start after enhanced >100 HU in the ascending aorta; 0.6 mm collimation) showed right apical condensations, a peribronchial nodule and a 7 mm pseudoaneurysm of the segmental apical artery of the right upper lobe (Figure 2b, 2c, 2d). Arteriography of the right pulmonary artery and selective catheterism of the upper right lobe segmental pulmonary artery allowed embolisation of the carrier pedicle of the RA with microcoils (Figure 2e, 2f). One week later, control CT confirmed occlusion of the RA.

## Discussion

We herein reported two cases of lung infection-related hemoptysis, respectively from bronchial and pulmonary origin, both identified on contrast-enhanced CT, prior to endovascular bleeding control.

CT and bronchoscopy are complementary tools for assessing patients with hemoptysis. The combination of CT and fibroscopy shows the cause of hemoptysis in 93% of cases, whereas fibroscopy alone shows it in 42% and CT alone in 67% [3]. Currently, in case of hemoptysis, bronchoscopy is still used as a first-line diagnostic and therapeutic tool, often helping to control bleeding. In the context of massive hemoptysis with ineffective bronchoscopic treatment, patients might benefit from angiography with embolisation when possible. Nevertheless, it is now established that CT is more effective than bronchoscopy in localizing the cause of hemoptysis [4,5]. Therefore, when the clinical condition of the patient allows, CT should be performed to guide angiography [6]. Furthermore, in 63–100% of patients with hemoptysis, CT can differentiate between systemic and pulmonary bleeding [4,5]. This may be of importance in the setting of infection-related hemoptysis as bronchial arteries are the source of bleeding in more than 90% of cases [7], presumably due to increased bronchial arterial flow and pressure and/or focal attenuation of the arterial wall strength [8,9]. Therefore, when the source of bleeding is not identified prior to angiography, a systemic catheterism is performed first, which may cost precious time when bleeding arises from the pulmonary artery, such as in Case 2.

In practice, in patients with hemoptysis, CT is performed in the systemic arterial phase. However, with the current scanner speed and low doses of contrast, a bolus of contrast that is too short may not allow visualisation of the pulmonary arteries. It is therefore advisable to use a larger bolus of contrast at the same rate (100 mL instead of 80 mL in our practice) so that both bronchial and pulmonary arteries are opacified upon scanning.

## Conclusion

CT usually allows the identification of the source of bleeding in lung infection-related hemoptysis and helps to plan the appropriate bleeding-control procedure.
